# Deciphering Electrochemical Generation, Transport, and Reactivity of Hydrogen Species in the Electrochemical Palladium Membrane Reactor

**DOI:** 10.1002/advs.78365

**Published:** 2026-10-18

**Authors:** Samuel J. Shin, Su Bin Roh, Hyun‐Tak Kim, Kwan‐Young Lee, Soo Min Kim, Ji Hoon Park, Minju Park

**Affiliations:** ^1^ CO_2_ & Energy Research Center Korea Research Institute of Chemical Technology (KRICT) Daejeon Republic of Korea; ^2^ Department of Chemical and Biological Engineering Korea University Seoul Republic of Korea; ^3^ Department of Advanced Materials and Chemical Engineering University of Science and Technology (UST) Daejeon South Korea

**Keywords:** electrochemistry, hydrogen atom, hydrogenation process, hydrogenation reaction, palladium hydride

## Abstract

Electrochemical palladium membrane reactor (ePMR) enables hydrogenation chemistry by mechanically separating, but (electro)chemically coupling reduction and chemical hydrogenation by transporting hydrogen species through a dense Pd membrane, yet the identity and transport mechanism of the reactive hydrogen species remain underdefined. Here, we disentangled the roles of hydrogen species, including hydrogen atoms and molecules for both electrochemical and chemical compartments using 1hexyne electrochemical hydrogenation reaction as a model reaction. Tafel analysis with H/D electrolytes, isotopic effects, and quantification of products show that the apparent kinetics were strongly affected by the electron transfer kinetics and hydrogen permeation through the Pd membrane. Furthermore, mathematical description of hydrogen atom transport through the Pd membrane provided qualitative model framework. Electrochemically deposited Pd, Rh, and PdRh layers with controlled thickness and morphology tuned the reaction pathway of hydrogen atom vs. H_2_‐mediation at the chemical compartment, enabling systematic variation of 1‐hexyne conversion and *n*‐hexane selectivity. A dominant role of in situ generation and re‐dissociation of H*/H_2_ was observed in the hydrogenation process, supported by control experiments including gas‐purging experiments. Collectively, these insights establish design principles of surface engineering for ePMR, strategically coupling atomic and molecular hydrogen pathways for efficient and selective organic hydrogenation.

## Graphical abstract

This study elucidates hydrogen transport and reaction mechanisms in ePMR using 1‐hexyne hydrogenation as a model reaction. Hydrogen transport operates via dual pathways involving hydrogen atom permeation and H_2_‐mediated re‐dissociation on secondary metal layers. Direct integration of secondary metals onto Pd_m_ minimizes the distance between permeated hydrogen species and secondary metal layers for H_2_‐mediation, providing a distinctive strategy for electrochemical hydrogenation.

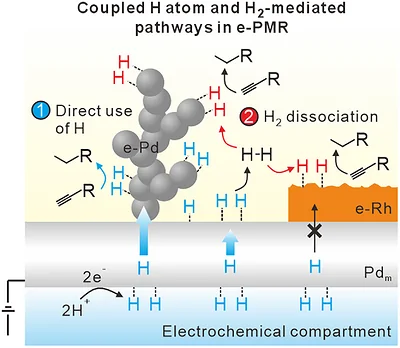

## 1 Introduction

Hydrogenation is a powerful chemical transformation reaction with widespread application in both academic research and industry for the synthesis of pharmaceuticals, functional materials, and fine chemicals, establishing it as a key reaction for advancing hydrogen economy [1]. In general, most traditional hydrogenation strategies are constrained by the requirements of high‐pressure molecular hydrogen (H_2_), elevated temperatures, transition metal catalysts, stoichiometric chemical reducing agents, and harsh reaction conditions [2, 3]. Furthermore, the solubility and mass transport of H_2_ are critical factors that often limit the catalytic performance and restrict the permissible reaction environment [4].

Electrochemical hydrogenation alternatively utilizes a renewable source of electricity to drive hydrogenation reactions and has been demonstrated in various applications, including the reduction of C = O and C = C bonds and biomass reforming [5, 6, 7, 8]. In these processes, protons are reduced to active hydrogen species at the catalytic site, which subsequently react with organic substrates. The necessary hydrogen species can be obtained from protic electrolytes (typically water), which offer a sustainable alternative to conventional hydrogenation processes that rely on gaseous H_2_ [5]. However, this method is subject to several inherent limitations. First, the reduction of protons must compete with the hydrogen evolution reaction (HER) at the cathode, potentially lowering the Faradaic efficiency. Second, aqueous electrolytes typically exhibit low solubility in organic molecules, restricting the scope of the available reactants. To address these limitations, several studies have investigated systems utilizing both aqueous and organic phases for the hydrogenation of organic reactants [9, 10]. For example, an organic hydride system involving the reduction of toluene to methylcyclohexane was explored using electrochemical water electrolysis. However, this approach faces severe practical limitations owing to two critical issues: first, the significant mass transport barrier created by the high interfacial tension between immiscible toluene (organic) and aqueous phases, and second, the risk of electrode damage and stability loss caused by toluene crossover on the electrode surface. This issue is not limited to toluene, but most water‐insoluble organic compounds present a challenge when applied to hydrogenation reactions driven by water electrolysis.

To circumvent these limitations, an electrochemical palladium membrane reactor (ePMR) has been developed and studied for several years (Scheme 1a) [11, 12, 13, 14, 15]. Unlike conventional electrochemical reactors, it physically segregates the electrochemical environment from the organic hydrogenation phase using a Pd membrane (Pd_m_), and the sections are termed as the electrochemical and chemical compartments, respectively. This configuration presents a key advantage of the decoupled reaction environment over the electrochemical environment—it allows hydrogenation to proceed efficiently in the pure organic phase without interfacial mass transport resistance, solubility issues of organic compounds in the electrochemical environment leading to multiphase electrochemistry, and the need for electrolyte purification in the chemical compartment, hydrogen solubility, or electrode fouling by organic species. Thus, ePMR offers a powerful platform for various chemical transformations, including C = C, C≡N, and C = O hydrogenation, deuteriation, and biomass reforming [16, 17, 18, 19]. The hydrogenation activity of the ePMR has been extensively studied using various secondary metals such as Pt, Ir, Au, and Rh [20, 21]. However, contemporary research on the ePMR has predominantly focused on the synthesis of active surfaces and reactant species, thereby lacking a cohesive understanding of the hydrogenation mechanism [8]. Few studies have elucidated the pathways for electrochemical hydrogenation via hydrogen species at the chemical compartment in terms of the chemical behavior of hydrogen species, hydride reactivity via a two‐combined electrolytic circuit, adsorption control of reactants, and by physically isolating Eley−Rideal and Langmuir–Hinshelwood pathways [22, 23, 24, 25, 26]. Still, in‐depth studies on the reactivity and reaction pathways occurring on secondary metals remain limited. Consequently, there is a rising scientific demand to clarify hydrogen behavior within the Pd_m_ and between reactants, which is challenging owing to the complex interactions between the active site and reactants. Moreover, the electrochemistry in the electrochemical compartment and elucidation of hydrogen transport through Pd_m_ have been relatively less recognized. This feature is important because it is chemically coupled to the chemical reactions occurring in the chemical compartment, which have been of interest in the literature, vide supra. Thus, addressing the above issues may provide directions for developing efficient reactors with enhanced hydrogenation performance to advance the utilization of the ePMR system.

**SCHEME 1:**
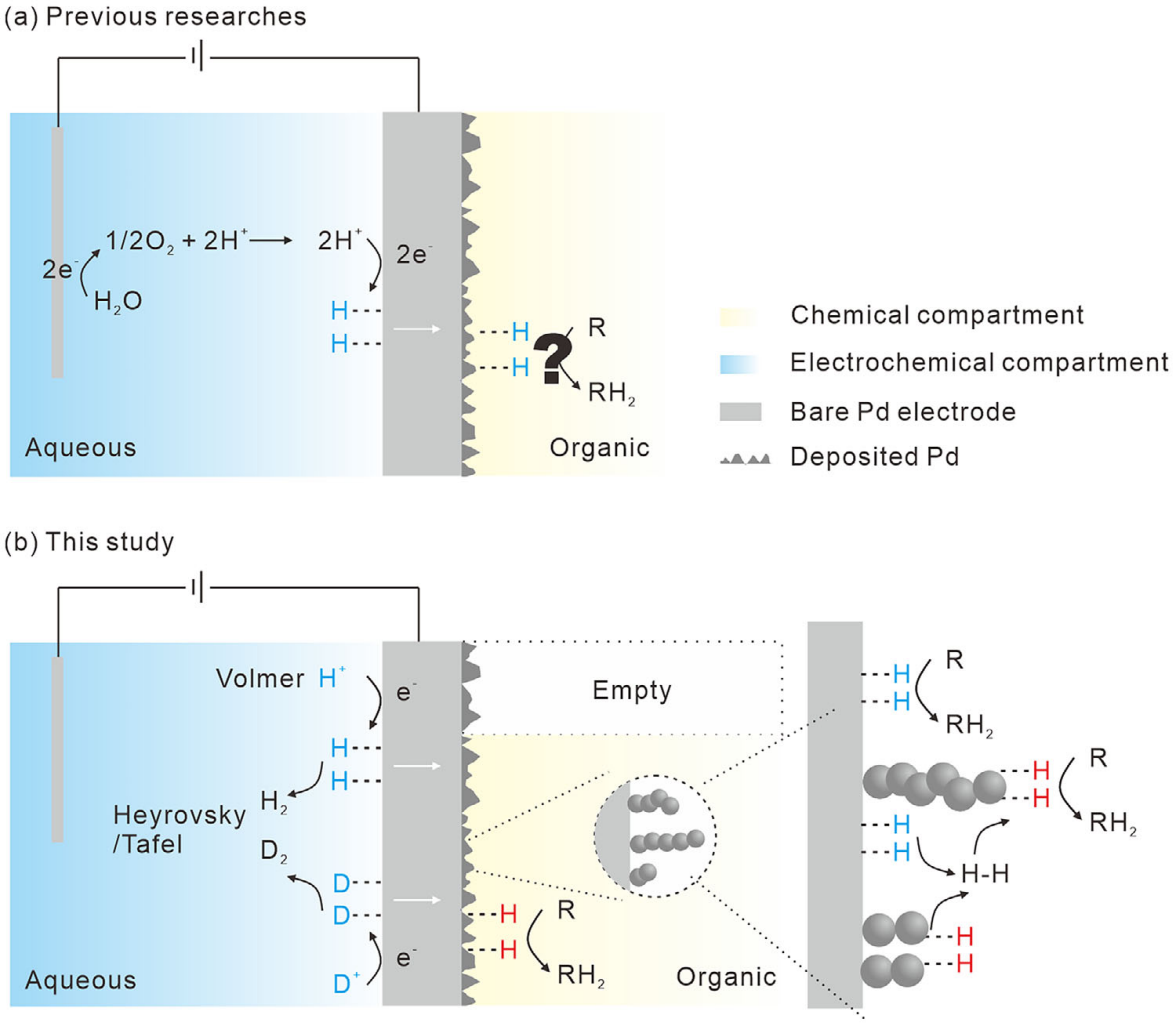
Comparative schematic illustration for (a) the conventional ePMRs reported in previous works and (b) the mechanistic objectives in the present study.

Herein, we performed an in‐depth investigation of the ePMR, utilizing the hydrogenation of 1‐hexyne as a model reaction to probe the fundamental processes. Our research specifically focused on the behavior of the hydrogen species and the underlying reaction mechanism within both the electrochemical and chemical compartments (Scheme 1b). Contrary to the prevailing assumptions, in which atomic hydrogen is considered the major reactive species, our results reveal that the behavior of the hydrogen species is significantly dependent on the morphology and thickness of the secondary metal layer. Crucially, we demonstrate that the involvement of hydrogen molecules becomes the predominant factor under specific conditions, and that the mass transport of H_2_ is also a critical factor affecting hydrogenation performance. These novel mechanistic insights provide a new perspective and approach for the rational design and optimization of the ePMR architecture.

## 2 Results and Discussion

### 2.1 Investigation of ePMR for Bare Pd_m_

A key aspect in ePMRs is the combination of electrochemistry and chemical reactions, which are mechanically separated by Pd_m_ but chemically coupled by hydrogen atoms. It can be inferred that the electrochemical properties of the electrochemical compartment depend on the chemical environment in the chemical compartment. Thus, the electrochemical kinetics of the ePMR with bare Pd_m_ were studied by employing Tafel studies and dividing the chemical compartment into three—empty (air), solvent only, and reactant dissolved in the solvent (Figure 1a,b). Note that the plots of Figure 1b were triplicated and drawn by average, and errors described in the following texts. The measured Tafel slope for the empty chemical compartment was 77±2 mV dec^−1^. Note that this value is lower than that of the typical Tafel slope observed for a Pd electrode immersed in a strongly acidic aqueous electrolyte of which has been well reported [27, 28]. The electron transfer kinetics observed for the ePMR may be attributed to an additional Tafel step at the empty chemical compartment (air) facilitated by permeated hydrogen atoms (path 2 in Figure 1a). A similar argument can be applied to the chemical compartment filled with solvent only, where the measured Tafel slope was 74±3 mV dec^−1^, similar to that of empty chemical compartment (air). The Tafel slope became even steeper to 83±4 mV dec^−1^ when the reactant was added, indicating that the overall electron transfer kinetics became sluggish. If the elementary step of 1‐hexyne hydrogenation at the interface of Pd_m_ in the chemical compartment is strongly favored, the depletion of hydrogen atoms at the electrochemical interface becomes dominant, which occurs due to the permeation/diffusion of hydrogen atoms through Pd_m_. Consequently, the contribution of the Volmer step was enhanced in the overall electron transfer kinetics of hydrogenation, relatively lowering the contribution of the Tafel/Heyrovsky steps. A similar trend in the Tafel slope change in the chemical compartment was also observed when the proton source was changed to deuterons. However, their Tafel slopes were significantly smaller than those of protons, implying that the contribution of Tafel/Heyrovsky steps was relatively more effective for the electron transfer kinetics in the deuterated electrolyte, as the slopes for the empty and solvent‐only compartments were 37±2 and 56±1 mV dec^−1^, respectively. Note that the diffusion of deuterium atoms through the octahedral holes of Pd_m_ may be sluggish compared to that of hydrogen atoms because of their lower solubility in Pd metal and larger size, impeding mass transport to the chemical compartment for hydrogenation [29]. Consequently, the deuterium gas evolution pathway for the electrochemically generated deuterium atoms may be strongly preferred in the electrochemical compartment, leading to preferred Tafel/Heyrovsky steps. Nevertheless, the Tafel slope of 66±2 mV dec^−1^ observed when the 1‐hexyne reactant was dissolved in the chemical compartment solution suggests that the contribution of the Volmer step became favorable when the mass transport equilibrium of deuterium atoms was enhanced by depletion in the chemical compartment.

**FIGURE 1:**
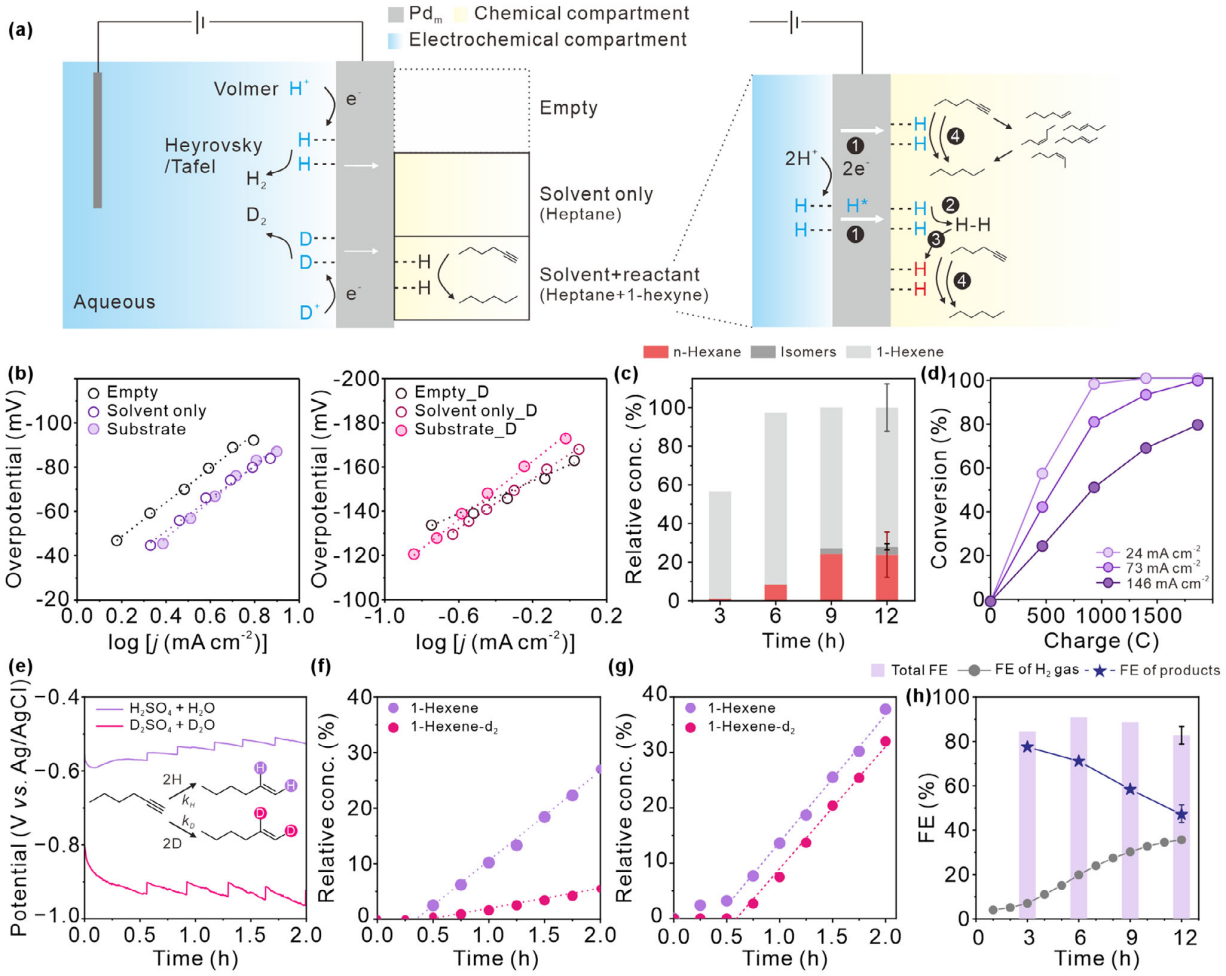
(a) Illustration of ePMR investigation for bare Pd_m_ by dividing it into three sectors for mechanistic study. (b) Tafel plots using proton (left) and deuteron (right) by varying the chemical compartment environment. (c) Product analysis of electrochemical hydrogenation of 1‐hexyne via ePMR of bare Pd_m_ in galvanostatic electrolysis and (d) conversion of 1‐hexyne by varying the applied current and charge. (e) Representative *V‐t* curve vs. Ag/AgCl reference electrode obtained for constant‐current electrolysis of 1‐hexyne by varying the proton sources of proton vs. deuteron. Kinetics study of the electrochemical hydrogenation of 1‐hexyne measured at the chemical compartment via (f) chronoamperometry (potentiostatically) and (g) chronopotentiometry (galvanostatically). (h) Quantification of evolved H_2_ gas at the chemical compartment. Relative conc. represents relative concentration.

Another important feature of ePMR is the diffusion of hydrogen atoms through the Pd lattices of the membrane via octahedral holes, which enables (electro)chemical coupling between two distinct (electro)chemical compartments. This mechanism has been extensively studied for hydrogen gas separation at high pressures and temperatures [30, 31, 32, 33]. However, the hydrogen atom diffusion mechanism is significantly different from that of hydrogen gas separation methods. This is because the origin of hydrogen species for hydrogen atom solubility differs from that of bulk Pd; specifically, electrochemically generated hydrogen atoms for ePMR differ from H_2_ gas in Pd_m_. Hence, in this study, the physical description of hydrogen atom diffusion through Pd_m_ was explored. The hydrogen gas flux for a thick Pd_m_ in the steady state has been well reported in many high temperature/pressure hydrogen gas separation studies, starting by thermochemical equilibrium of chemical potentials of H_2_ and hydrogen atoms dissolved in Pd lattices (H_Pd_), $\mu_{H_{2}}=2\cdot \mu_{H_{\mathrm{Pd}}}$, further derivation of Sievert's law and combination with Fick's first law (Equation 1) [34].

(1) $$J_{H_{2}}=\frac{D_{H_{2}}\cdot k_{S,\;H_{2}}}{L}\;\cdot \left(\sqrt{P_{H_{2},feed}}-\sqrt{P_{H_{2},perm}}\right)$$

where $J_{H_{2}}$ is the hydrogen gas flux, $D_{H_{2}}$ is the diffusion constant of hydrogen gas, $k_{S,\;H_{2}}$ is Sievert's constant, *L* is the thickness of Pd_m_, and $P_{H_{2},feed}$ and $P_{H_{2},perm}$ are the partial pressures of the fed and permeated hydrogen gas, respectively. Further, the term $D_{H_{2}}\cdot k_{S,\;H_{2}}$ represents the permeability of hydrogen gas. Note that the square‐root term originates from both the 1:2 stoichiometry between H_2_ gas and H_Pd_ and the expression of activities out of the logarithmic term, of which activities are converted to dimensionless partial pressures.

For ePMR, the physical meaning of the diffusion flux refers to the hydrogen atoms. The chemical potentials may be written by the balance of adsorbed hydrogen atoms (H*) and H_Pd_, $\mu_{H^{*}}=\mu_{H_{\mathrm{Pd}}}$. Thus, Equation (1) can be adapted to Equation (2) by utilizing the following electrochemical parameters without square roots:

(2) $$J_{H^{*}}=\frac{D_{H^{*}}\cdot k_{S,\;H^{*}}}{L}\;\cdot \left(a_{H^{*},feed}-a_{H^{*},perm}\right)$$

where the terms are similar, but the physical definitions are referred to as H* whether fed or permeated, and pressure is substituted for the activity of H*, $a_{H^{*}}$. Note that the state of PdH_x_ generated by the diffusion of hydrogen atoms in bulk Pd was unnecessary to be described due to the steady‐state assumption.

By adapting a simple mass balance, the flux of hydrogen species in the ePMR can be described as follows (Equation 3):

(3) $$\frac{i}{\mathrm{FA}}=J_{H^{*}}+2\cdot J_{\mathrm{HER}}$$

where *i* is the current, F is the Faraday constant, A is the effective area corresponding to both the electrode surface exposed to the solution and hydrogen transport to Pd_m_, and *J*_HER_ is the flux of H_2_ upon the HER (corresponding to the actual gas molecule generated) in the electrochemical compartment. Note that this mass balance assumes a galvanostatic mode (constant current) and a unity of Faradaic efficiency (FE) for the electrochemical generation of hydrogen species. By combining Equations (2 and 3), the following Equation 4 is developed:

(4) $$J_{H^{*}}=\frac{i}{\mathrm{FA}}\;-2\cdot \;J_{\mathrm{HER}}=\frac{D_{H^{*}}\cdot k_{S,\;H^{*}}}{L}\;\cdot \left(a_{H^{*},feed}-a_{H^{*},perm}\right)$$

The time‐dependent form (quasi‐steady‐state) is expressed as follows:

(5) $$\frac{dn_{H^{*}}}{dt}=\frac{i-i_{\mathrm{HER}}}{F}\;-\frac{D_{H^{*}}\cdot k_{S,H^{*}}\cdot A}{L}\cdot \left(a_{H^{*},feed}-a_{H^{*},perm}\right)$$

where $n_{H^{*}}$ is the number of moles of H* near the electrode, and *i*_HER_ is the partial current of the HER.

Note that this model provides a useful qualitative framework to understand the phenomena occurring at the Pd_m_, but is limited without complete validation. For instance, *D* and *k*_S_ values for H* have been underexplored compared to those for H_2_. We believe that computational chemistry, including DFT calculations or MD simulations, can further decipher the two characteristic parameters for mechanistic investigations. Furthermore, proper COMSOL/CFD simulations based on the models provided above may support transport phenomena of such an electrochemical system in the near future.

The final key aspect of ePMR is the hydrogenation reaction occurring in the chemical compartment via the electrochemically generated hydrogen atoms in the electrochemical compartment. Note that both the thermodynamics and kinetics of hydrogenation differ significantly depending on which hydrogen species react with the reactant, that is, protons, hydrogen atoms, or hydrides, but this has been less studied. For example, Stankovic et al. suggested that the chemical behavior of hydrogen species in an ePMR is strongly affected by the applied current density, tuning the reactivities of the three hydrogen species of protons, hydrogen atoms, and hydrides [22]. Similarly, Hu et al. reported hydride reactivity by employing two electrolytic circuits with Janus Pd_m_ as the cathode [23], Moreover, Li et al. and Zhang et al. reported deuterium isotopic labeling study to confirm the source of hydrogen originated from the electrochemical compartment and elucidated reaction pathways such as adsorption of reactant, Eley−Rideal pathway, and Langmuir‐Hinshelwood pathway for hydrogenation at the chemical compartment [24, 25]. To advance the understanding of the chemical nature of this system, mechanistic study on the reaction kinetics and reaction intermediates must be further conducted to fill the missing piece of information.

We first conducted a product analysis of 1‐hexyne hydrogenation via ePMR by applying a constant current density of 24 mA cm^−2^ (Figure 1c and Figure S1). It must be mentioned that 1‐hexyne reactant was not fully converted until 3–6 h of electrolysis, and its depiction of bar graph for relative concentration was omitted. Please refer to the Experimental Section for quantitative definitions of relative concentration, composition, conversion, and FE throughout this work. In addition, standard conditions were chosen based on screening of solvent and applied potentials, followed by the choice of galvanostatic mode (Figure S2). Although stepwise hydrogenation of 1‐hexyne to hexene followed by further hydrogenation to *n*‐hexane occurred during the time‐course electrolysis, hexene selectivity was dominant and was comparable to that reported in the literature [16, 35]. This indicates the hampered kinetics for the full alkyne‐to‐alkane hydrogenation of ePMR on Pd_m_ but does not clarify the dominant hydrogen species for hydrogenation reactivity. Thus, we revisited the conversion efficiency of 1‐hexyne by varying the applied current density and accumulated charge (Figure 1d and Figure S3), similar to that applied in a previous study on the electrochemical hydrogenation of TEMPO to TEMPO‐H via ePMR [22]. It was found that lower current density efficiently achieved 1‐hexyne hydrogenation compared to higher current densities. In a previous report, hydrogen atom reactivity toward TEMPO, proton/hydride reactivity toward the pH of the solution, and NADH electrochemistry suggested that a lower current density was governed by hydrogen atoms and a higher current density by proton/hydride reactivity [14, 22]. Thus, it can be inferred that the hydrogen atom reactivity of ePMR was favorable for 1‐hexyne hydrogenation. This was further confirmed by investigating the rate studies and apparent isotopic effect of electrochemical hydrogenation in the chemical compartment via ePMR for bare Pd_m_ in both galvanostatic and potentiostatic modes (Figure 1e–g and Figure S4). Based on the potentiostatically measured isotopic ratio of 4.5 implied that the contribution of proton transfer step was significant, which agrees well with the enhanced contribution of Volmer steps based on the Tafel analysis discussed above [36]. A galvanostatically measured rate study showed that the hydrogenation/deuteration reaction rates were almost identical in the chemical compartment, implying that the reaction rate was strongly proportional to the electrochemical reaction rate in the electrochemical compartment for H* generation. The time delay of the reaction initiation between protons and deuterons may be due to the sluggish mass transport of deuterium atoms compared to that of hydrogen atoms, substantially contributing to a higher overpotential for the same applied current. The main side reaction was hydrogen gas evolution via the Tafel pathway in the chemical compartment, where the quantity of hydrogen gas was quantified (Figure 1h).

### 2.2 Characterizations of Electrochemically Deposited Pd_m_ and Its Electrochemical Hydrogenation

Based on an in‐depth study of ePMR using bare Pd_m_, we further investigated its hydrogenation performance in 1‐hexyne conversion as a function of the secondary metal layers and their morphologies. Herein, we systematically investigated the dependence of the hydrogenation reaction by electrochemically depositing Pd, Rh, and bimetallic Pd‐Rh onto Pd_m_ surfaces (Figure 2a). The reduction potentials for electrodeposition were determined using linear sweep voltammetry (LSV), as presented in Figure 2b. Two distinct reductive potential peaks at +0.15 V and −0.15 V were selected for the electrochemical deposition process. The analysis of the secondary metal layer via scanning electron microscopy (SEM) revealed that Pd deposition was achieved at both +0.15 V and −0.15 V (Figure S5), resulting in samples designated as e‐Pd/Pd_m_. Conversely, a substantial Rh coating was realized at +0.15 V, yielding e‐Rh/Pd_m_, whereas insufficient Rh layer formation was evident at −0.15 V (Figure S6). Moreover, X‐ray photoelectron spectroscopy (XPS) spectra of the electrodeposited layers on Pd_m_ confirmed that metallic Pd (Pd^0^) was successfully developed at both +0.15 V and −0.15 V (Figure S7). In contrast, when Rh electrodeposition was prepared at −0.15 V, the Rh species was almost entirely in the oxidized state (Rh^3+^) rather than the desired metallic Rh (Rh^0^) form (Figure S8). We presume that the local pH increased at the Pd_m_ surface via proton reduction occurred at −0.15 V, preferentially forming oxide or hydroxide surfaces on Rh based on Pourbaix diagram [37, 38]. Based on these findings, +0.15 V was selected as the optimal potential to fabricate all secondary metal layers used in this study, including monometallic e‐Pd/Pd_m_ and e‐Rh/Pd_m_, as well as the co‐deposited bimetallic sample, designated as e‐PdRh/Pd_m_ (Figures S9 and S10). Figure 2c illustrates the distinct morphologies of the deposited layers on Pd_m_ as observed by SEM. e‐Pd/Pd_m_ and e‐PdRh/Pd_m_ exhibited characteristic dendrite‐like features, in contrast to e‐Rh/Pd_m_, which formed a dense and thick layer on Pd_m_. Cross‐sectional scanning transmission electron microscopy (STEM) images and energy‐dispersive X‐ray spectroscopy (EDX) mapping collectively confirmed the successful deposition of both Pd and Rh on Pd_m_ and verified their divergent surfaces and cross‐sectional morphologies (Figure 2c and Figure S11). Interestingly, e‐Rh/Pd_m_ formed a notably thick Rh layer, exceeding 200 nm, which was specifically designed to restrict the penetration of hydrogen atoms through the metal lattices, which will be further discussed vide infra.

**FIGURE 2:**
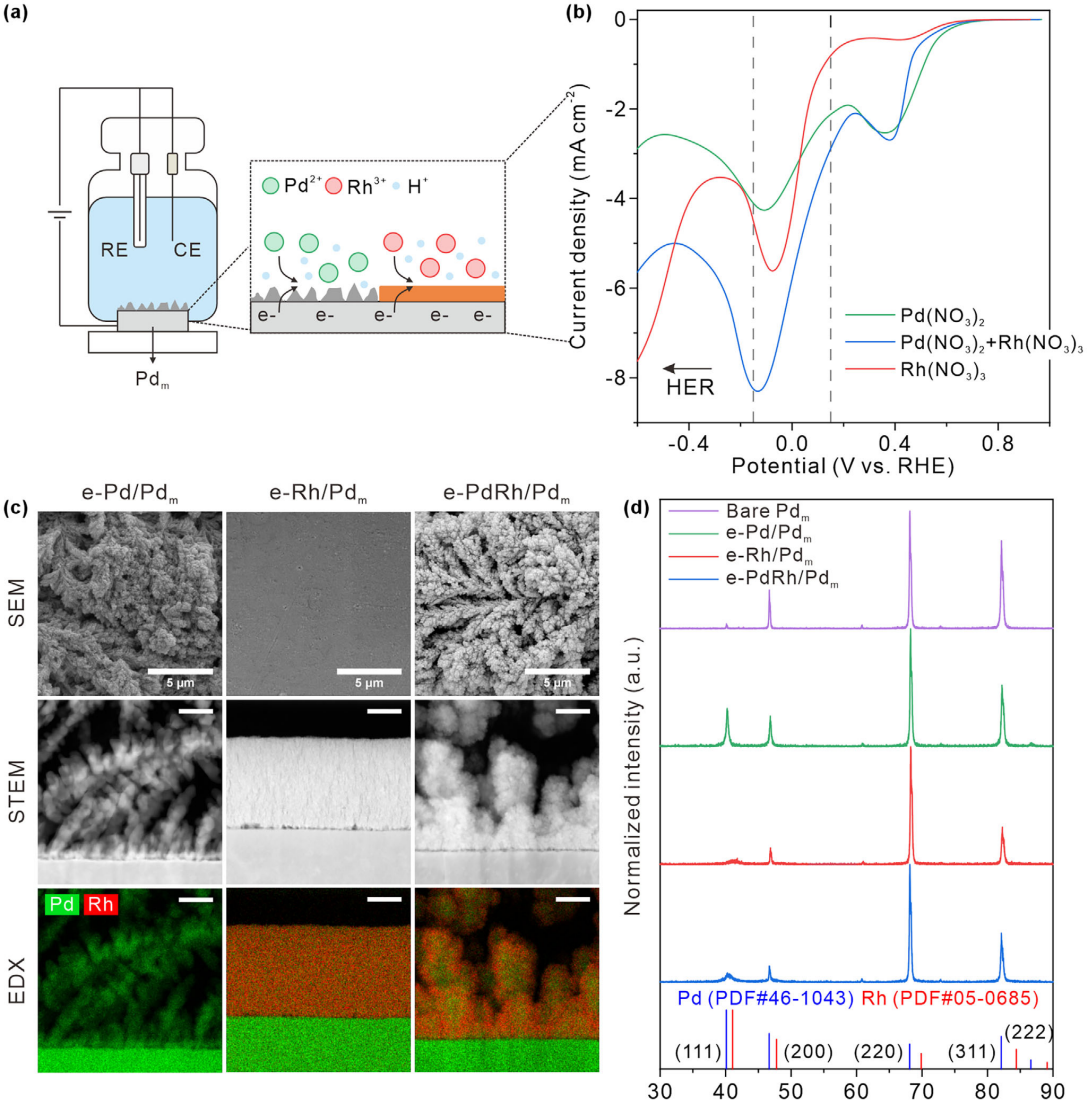
Preparation and characterization of the electrodeposits supported on Pd_m_. (a) Schematic illustration of the electrochemical metal deposition process. (b) LSV curves using different metal precursors. (c) SEM, cross‐sectional STEM images, and EDX mapping images of e‐Pd/Pd_m_, e‐Rh/Pd_m_, and e‐PdRh/Pd_m_. The scale bar for STEM and EDX mapping represents 50, 100, and 50 nm from left to right, respectively. (d) XRD patterns of bare Pd, e‐Pd/Pd_m_, e‐Rh/Pd_m_, and e‐PdRh/Pd_m_.

X‐ray diffraction (XRD) was used to investigate the crystal structure. Bare Pd_m_ exhibited three characteristic peaks at 2θ values of 46°, 68°, and 82°, corresponding to the (200), (220), and (311) crystalline planes of face‐centered cubic (fcc) Pd, respectively (Figure 2d). e‐Pd/Pd_m_ displays an additional prominent peak at 40°, which corresponds to the (111) crystalline plane of fcc Pd [39]. When the Pd particles deposited onto Pd_m_ were analyzed separately and designated as e‐Pd, the (111) peak near 40° was the most prominent feature (Figure S12). This result confirmed that the specific peak at 40° originates from the e‐Pd layer. In addition, e‐Rh/Pd_m_ exhibits additional characteristic peaks at approximately 41°, corresponding to the (111) crystalline plane of Rh.

Electrochemical hydrogenation of 1‐hexyne using the fabricated Pd_m_ was also investigated. The concentrations of reactants and products were determined by gas chromatography‐mass spectrometry (GC‐MS) under a constant current density of 24 mA cm^−2^ (Figure S13). For the bare Pd_m_, 1‐hexyne was fully converted after 12 h, generating *n*‐hexane at a relative concentration of 24% (Figure 1d). Following the electrochemical deposition of Pd onto the Pd_m_, the resulting e‐Pd/Pd_m_ exhibited a significantly enhanced hydrogenation performance for the conversion of 1‐hexyne. This membrane achieved a relative concentration of *n*‐hexane exceeding 88% within 12 h (Figure 3a and Figure S14a). The bimetallic e‐PdRh/Pd_m_, prepared with a surface morphology similar to that of e‐Pd/Pd_m_, also demonstrated efficient 1‐hexyne conversion within 12 h (Figure 3c and Figure S14c), achieving a 95% relative concentration of *n*‐hexane.

**FIGURE 3:**
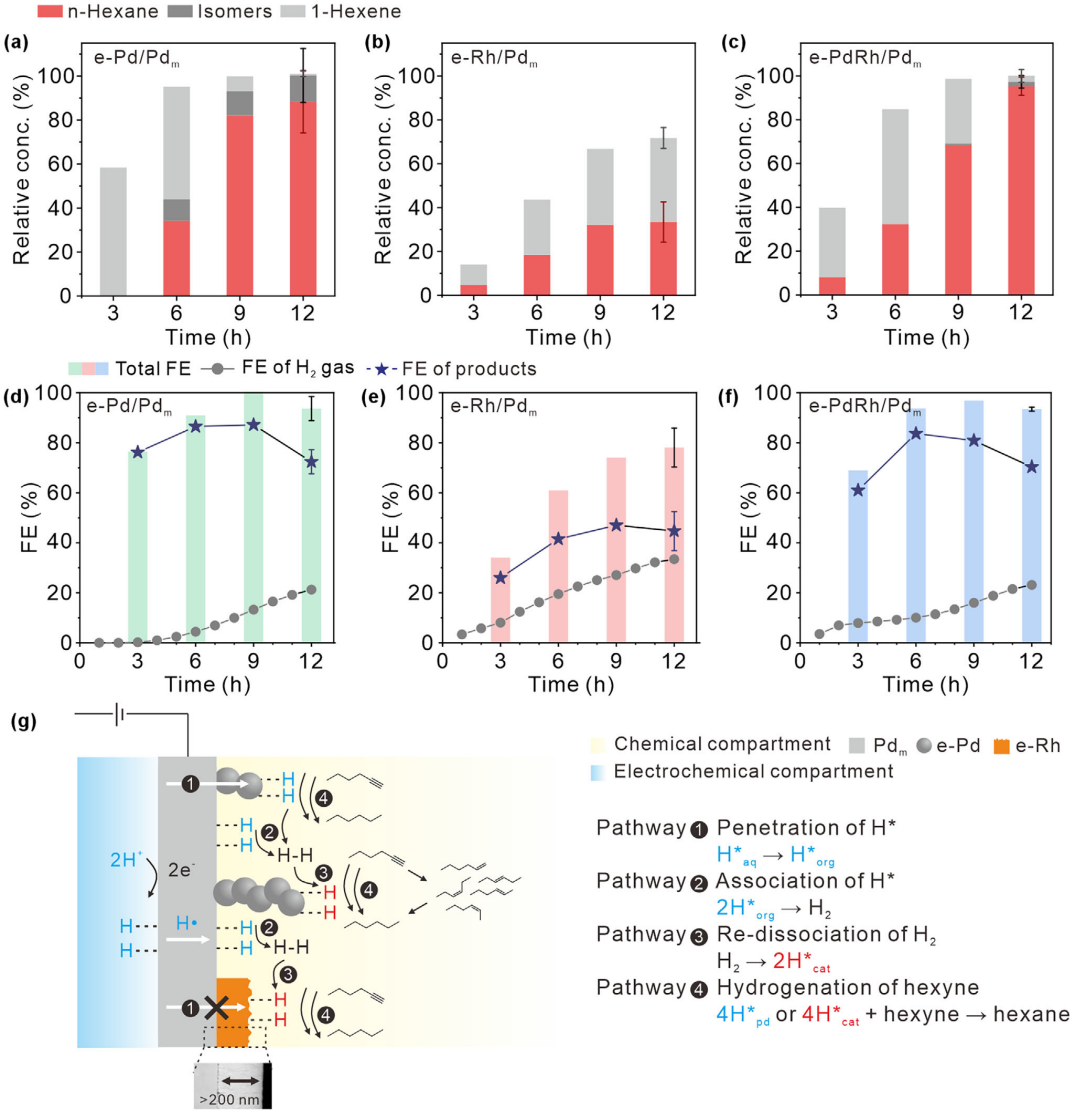
Hydrogenation performance evaluation during a 12 h reaction period using (a) e‐Pd/Pd_m_, (b) e‐Rh/Pd_m_, and (c) e‐PdRh/Pd_m_. FE of H_2_ gas and *n*‐hexane production using (d) e‐Pd/Pd_m_, (e) e‐Rh/Pd_m_, and (f) e‐PdRh/Pd_m_. (g) Schematic illustration of the proposed hydrogenation mechanism during 1‐hexyne conversion in the ePMR. Relative conc. represents relative concentration.

While previous studies have proposed that the primary mechanism for hydrogenation in an ePMR involves the permeation of hydrogen atoms through both the Pd_m_ and the electrodeposited secondary metal layers, our experiments suggest the existence of an additional major pathway for the hydrogenation of 1‐hexyne. A critical observation supporting this alternative mechanism is that hydrogenation proceeded even under conditions in which hydrogen atom permeation is markedly restricted by the electrochemically deposited Rh layer in e‐Rh/Pd_m_. This may be inferred from the fact that hydrogen atoms cannot permeate through interstitial sites of Rh in ambient condition [40]. Despite the presence of a relatively thick Rh layer, which was expected to impede hydrogenation, the e‐Rh/Pd_m_ system still exhibited a higher‐than‐anticipated 1‐hexyne conversion of 73% and an *n*‐hexane relative concentration of 33% at 12 h (Figure 3b and Figure S14b). This observation suggests that surface‐mediated hydrogenation pathways, rather than H* supplied from H_Pd_ via Pd_m_ permeation to the chemical compartment, play a predominant role in the reactivity. Besides, Rh is a well‐known catalytic material enabling catalytic hydrogenation of 1‐hexyne at ambient condition [41, 42]. Herein, we propose an additional plausible mechanism involving molecular hydrogen, H_2_. Following the reduction of protons on the aqueous‐side Pd surface (electrochemical compartment), the resulting H* permeate the Pd lattices of Pd_m_ in the form of H_Pd_ to the other side (chemical compartment), resulting H* at the surface. Once at the Pd_m_ surfaces in the chemical compartment, these hydrogen atoms can react via two potential pathways: they can either diffuse through the deposited Pd layer (Figure 3g, pathway 1) or associate with H_2_ molecules (Figure 3g, pathway 2) by combining with another H* (Tafel step at the chemical compartment). H* can directly hydrogenate 1‐hexyne (pathways 1 and 4), the hydrogen atom pathway (H atom pathway), a mechanism previously proposed for hydrogenation in ePMRs [13, 20]. Based on the discussion above, we hypothesized that the H_2_ generated via pathway 2 may re‐dissociate on the deposited secondary metal layers (Figure 3g, pathway 3) and subsequently contribute to the catalytic hydrogenation of 1‐hexyne (Figure 3g, pathway 4), providing an additional hydrogenation route, a molecular hydrogen‐mediated pathway (H_2_‐mediated pathway), that complements the H atom pathway. To investigate the major reaction pathways, H_2_ gas within the organic compartment was monitored by gas chromatography (GC). For the e‐Pd/Pd_m_, H_2_ gas was not detected until 3 h, indicating that most of the permeated hydrogen atoms were efficiently consumed by the hydrogenation of 1‐hexyne (Figure 3d). In sharp contrast, for the e‐Rh/Pd_m_ and e‐PdRh/Pd_m_ (Figure 3e,f), H_2_ gas was detected at an early stage of the reaction, strongly suggesting that molecular hydrogen may also contribute to the hydrogenation process in these systems.

### 2.3 Deciphering Hydrogenation of 1‐Hexyne With H_2_ Gas

To support our hypothesis regarding the involvement of H_2_‐mediated pathway, we investigated the hydrogenation of 1‐hexyne under H_2_ gas purging conditions (Figure 4a,g). Figure 4b shows the relative concentrations of the final products after 6 h hydrogenation of 1‐hexyne with H_2_ gas purging without electrochemical reaction. Interestingly, even with the bare Pd_m_, the 1‐hexyne conversion was 36% (Figure S15a). For e‐Pd/Pd_m_, 1‐hexyne was fully converted within 6 h with a *n*‐hexane relative concentration of 58%. e‐Rh/Pd_m_ exhibited a conversion of 31% and a *n*‐hexane relative concentration of 12%. These results suggest that H_2_ participates in the hydrogenation of 1‐hexyne. Specifically, the deuterium experiments (Figure 1f) confirmed that the protons used in the ePMR originated from electrochemical generation and subsequent permeation through the Pd_m_. This led to the assumption that a significant contribution to the reaction involves H_2_ diffusing into the organic compartment followed by re‐dissociating on the deposited metal surface to participate in the hydrogenation reaction.

**FIGURE 4:**
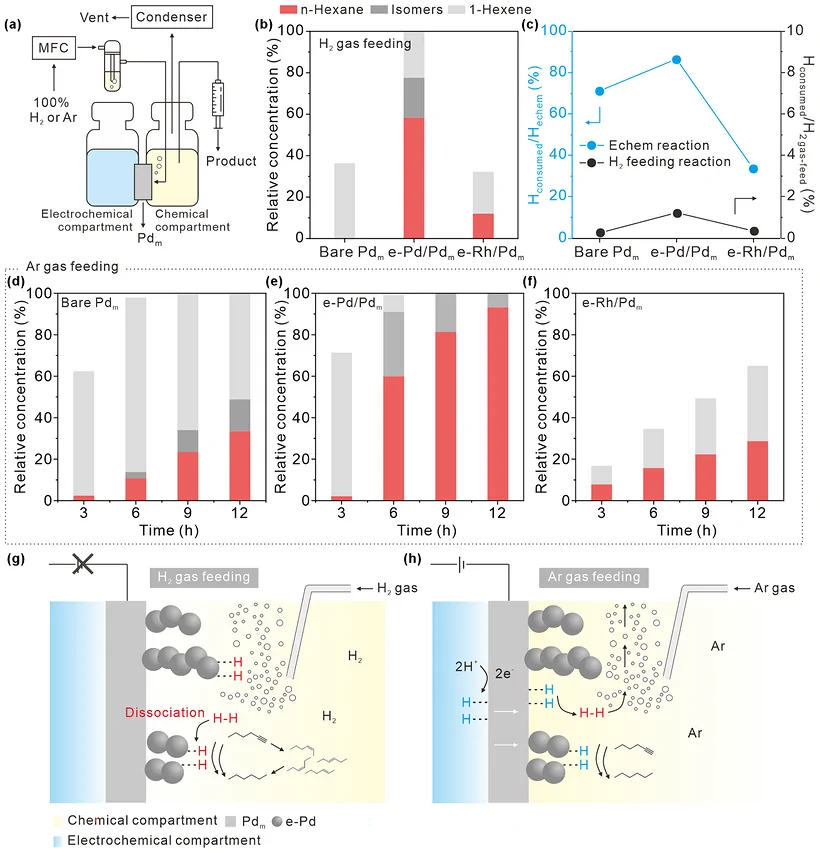
(a) Schematic illustration of the gas‐purging reactor setup used for the controlled experiments. (b) Relative concentration of 1‐hexyne conversion and *n*‐hexane production after the hydrogenation reaction under external H_2_ gas feeding. (c) Ratio of consumed hydrogen for comparing the electrochemical reaction (without external H_2_) and the H_2_ gas feeding reaction. Relative product concentration after 1‐hexyne hydrogenation under Ar gas purging using (d) bare Pd_m_, (e) e‐Pd/Pd_m_, and (f) e‐Rh/Pd_m_. Schematic illustration detailing the experimental conditions for (g) H_2_ gas feeding and (h) Ar gas feeding.

To provide further qualitative evidence of the role of H_2_ in the hydrogenation reaction, an Ar gas purging experiment was conducted (Figure 4h). If the production of H_2_ gas is indeed a critical factor, its physical removal by bubbling Ar to the solutions in chemical compartment should have a noticeable impact on the hydrogenation performance. In the case of bare Pd_m_, the relative concentration of *n*‐hexane was approximately 34% after 12 h and remained nearly unchanged regardless of Ar purging (Figure 4d). For the e‐Pd/Pd_m_, Ar purging resulted in a slight change in the relative concentration of *n*‐hexane, indicating a shift in the reaction selectivity (Figure 4e). This limited effect implies that the H‐atom pathway may be the dominant pathway in these systems, even though secondary Pd layer provides excellent catalytic hydrogenation sites for 1‐hexyne with H_2_ (Figure 4b). In contrast, the effect was much more pronounced for e‐Rh/Pd_m_, where Ar purging reduced the 1‐hexyne conversion from 73% to 68% and *n*‐hexane relative concentration decreased from 33% to 29% (Figure 4f and Figure S15b). These findings indicate that e‐Rh/Pd_m_ relied relatively more on H_2_‐mediated pathway than bare Pd_m_ or e‐Pd/Pd_m_ and was therefore more sensitive to perturbations in the reaction gas environment, with Ar purging substantially suppressing both the activity and selectivity for *n*‐hexane production. Taken together, these findings suggest that when hydrogen atom permeation through the metal lattice is facile at the chemical compartment (bare Pd_m_ and e‐Pd/Pd_m_), H_2_ plays only a minor role. On the contrary, when permeation is hindered at the secondary metal layer, such as in e‐Rh/Pd_m_, H_2_ in situ generation (Tafel step), re‐dissociation, and subsequent participation in hydrogenation constitute additional reaction pathways.

In conventional hydrogenation reactions, optimization of the hydrogenation performance is strongly influenced by the mass transport of H_2_ [43, 44]. Consequently, reactor design and architecture are crucial for maximizing hydrogenation efficiency in the field. From this perspective, the involvement of H_2_ in ePMRs may be pivotal. In the ePMR design, H_2_ may be evolved in close proximity to a Pd_m_. The architecture of direct contact of Pd_m_ with the electrodeposited metal layer thus facilitates the re‐dissociation of the H_2_ for further catalytic hydrogenation. Such a unique configuration allows for a more efficient hydrogenation performance when using electrochemically generated hydrogen atoms from abundant water compared to simply supplying H_2_ from an external source. This suggests that localized in situ generation and re‐dissociation of H_2_ may be more effective strategies for enhancing reactivity and performance.

To investigate the effect of H_2_ mass transport, the spatial arrangement of the Pd particles (e‐Pd, secondary Pd layer detached from e‐Pd/Pd_m_) was systematically controlled, as illustrated in Figure 5 and Figure S16. When the e‐Pd particles were dispersed in the chemical compartment (dispersed e‐Pd), the relative concentration of *n*‐hexane decreased from 97% to 74% compared with the standard configuration. A more significant reduction in performance was observed when the e‐Pd/Pd_m_ was physically separated from the bare Pd_m_ by a distance of approximately 0.5 cm (Pd+e‐Pd). In this configuration, the hydrogenation performance of *n*‐hexane decreases dramatically to a relative concentration of 34%. These results clearly demonstrate that the proximity of the secondary metal to the source of hydrogen species is a critical factor and that physical separation severely hinders the efficiency of the hydrogenation reaction owing to mass transport limitations.

**FIGURE 5:**
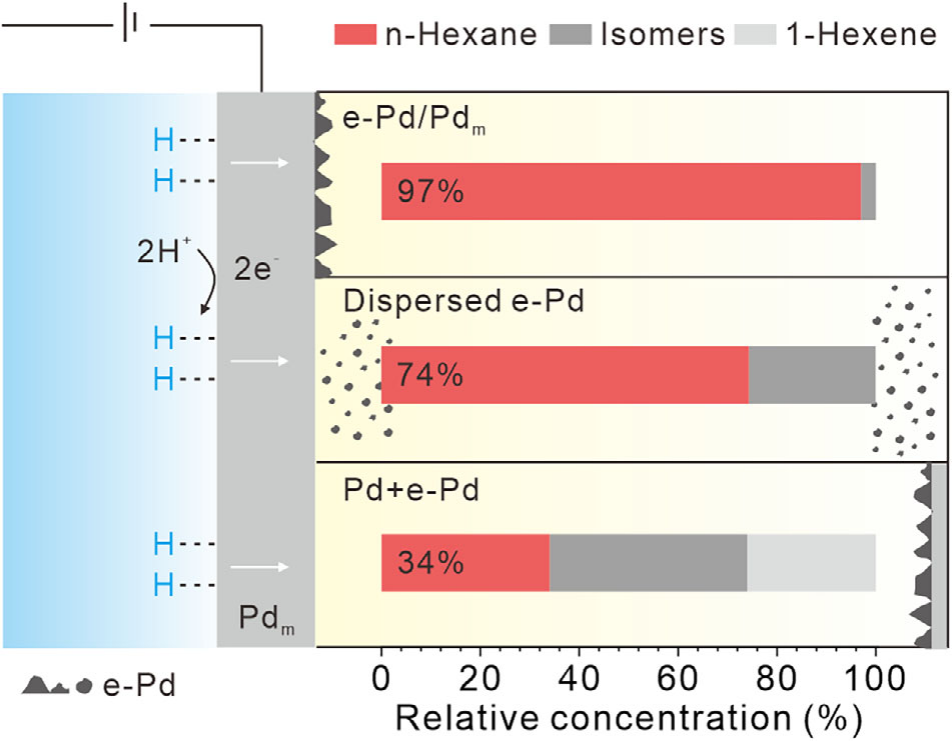
Schematic illustration of various ePMR designs and their corresponding hydrogenation performance.

Taken together, ePMR offers a significant mass transport advantage over conventional organic‐phase hydrogenation processes, particularly when H_2_‐mediated pathway is involved in the reaction. In the chemical compartment of ePMR, hydrogen atoms are supplied to the close proximity distance to the architecture of Pd_m_ and the secondary metal layer. This proximity inherently reduces the diffusion limitations associated with the transport of H* and 1‐hexyne for the reaction via H atom pathway. For H_2_‐mediated pathway, H_2_ overcome such diffusion limitation, thereby enhancing the mass transport efficiency of the catalytic reaction. Building on these mechanistic insights, ePMR is anticipated to advance into architectures incorporating diverse metal–catalyst configurations to further optimize/maximize their performance.

## 3 Conclusions

This study elucidated the fundamental hydrogen transport and reaction mechanisms in the ePMR through investigation of 1‐hexyne hydrogenation as a model reaction. By integrating electrochemical kinetics with mass transport models, we demonstrated that the ePMR operates through a complex interplay of two interfacials (electrochemical/chemical compartments) and bulk processes among Pd_m_. Tafel analysis and apparent isotopic studies identified that the increase of the Volmer step contribution was essential, while the overall hydrogenation rate was found to be strictly proportional to the electrochemically generated H* flux. The adaptation of Sievert's and Fick's laws provided a qualitative macroscopic physical description of hydrogen transport in the steady‐state. Such provided implication that efficient hydrogenation process at the chemical compartment facilitated the overall hydrogen transport, which was further confirmed that surface morphology and secondary metal composition dictate the dominant reaction pathway occurring at the chemical compartment. Through a systematic combination of electroanalytical characterization, controlled secondary metal layer electrodeposition, and mass transport investigations, we demonstrated that the reactivity of hydrogen species supplied to the chemical compartment do not follow a single dominant mechanism. Instead, hydrogenation was driven by the synergistic interplay of H atom pathway and H_2_‐mediated pathway. Specifically, the fabrication of e‐Pd/Pd_m_ and e‐PdRh/Pd_m_ significantly enhanced reactivity compared to the bare Pd_m_. More importantly, our results challenged the conventional H atom pathway; the high activity observed on e‐Rh/Pd_m_, despite its role of Rh as a barrier for permeation through its lattices, providing qualitative evidence for a H_2_‐mediated pathway. The substantial suppression of *n*‐hexane production upon Ar purging further suggests the contribution of H_2_ reactivity and mass transport. Finally, spatial arrangement of secondary metal layers investigation revealed that physical separation of the secondary metal layer led to a dramatic performance decrease, demonstrating that the hydrogenation efficiency was highly sensitive to the proximity distance between the secondary metal layer and Pd_m_. This underscores that the ePMR possesses an outstanding spatial advantage for hydrogenation, as the secondary metal layer where the chemical reaction occurs is directly integrated with the Pd_m_ from which atomic hydrogen permeates, thereby ensuring an immediate reaction sequence. Collectively, these findings provide mechanistic insights for the rational design of next‐generation ePMR systems for sustainable chemical synthesis.

## 4 Experimental Section/Methods

### 4.1 Materials

Palladium foil (0.025 mm thick, 99.9%) and platinum gauze (52 mesh, 99.9%) were purchased from Thermo Fisher Scientific. Sulfuric acid (ACS reagent, 95.0%–98.0%), sulfuric acid‐d_2_ solution (96–98 wt.% in D_2_O, 99.5 atom% D), 1‐hexyne (97%), 1‐hexene (≥99%), *trans*‐2‐hexene (97%), *trans*‐3‐hexene (≥99%), *n*‐hexane (ReagentPlus, ≥99%), *n*‐heptane (ReagentPlus, 99%), *n*‐pentane (CHROMASOLV, for HPLC, ≥99%), and bibenzyl (ReagentPlus, 99%) were obtained from Sigma‐Aldrich. Deuterium oxide (99.8%, isotopic), *cis*‐2‐hexene (96%), *cis*‐3‐hexene (97%), and platinum wires (0.5 mm, 99.997%) were purchased from Alfa Aesar. Palladium (II) nitrate hydrate (Pd ≥40%), and rhodium (III) nitrate (solution in water, 10% Rh) were obtained from STREM. Nitric acid (60.0%, extra pure), hydrogen peroxide (30.0–35.5%, special grade), and sodium hydroxide solution (1 N) were purchased from Samchun Chemicals. *n*‐Decane (extra pure, GC assay ≥95.0%) was purchased from Junsei. All the chemicals were used as received without further purification.

### 4.2 Electrochemical Characterizations

The electrochemical measurements were performed using a Bio‐Logic SP‐300 potentiostat. A Pd_m_ was used as the working electrode, a Pt mesh as the counter electrode, and Ag/AgCl (saturated KCl solution) as the reference electrode. All potentials in this work are reported vs. the reversible hydrogen electrode, RHE (otherwise stated), and converted using the equation below:

$$\;E_{\mathrm{RHE}}=E_{\mathrm{Ag}/\mathrm{AgCl}}+0.1951+\left(0.0591\times \mathrm{pH}\right)$$

All electrochemical measurements were performed in an electrochemical cell, which was constructed similarly to the H‐type cell with a Pd_m_ placed between the organic and aqueous compartments (unless otherwise stated). The geometric surface areas of the Pd_m_ were 1.77 cm^2^. Cyclic voltammograms (CV) were acquired in an aqueous compartment containing 35 mL of 1 M H_2_SO_4_ and the organic compartment was kept empty, with both compartments left unstirred. For the Tafel slope analysis, the aqueous compartment contained 35 mL of 1 M H_2_SO_4_ or D_2_SO_4_ in D_2_O for the isotope experiments, whereas the organic compartment was kept empty or contained 20 mL of heptane with or without 100 mM 1‐hexyne. Chronoamperometry (CA) was performed using an aqueous phase that was left unstirred, while the organic phase was stirred at 300 rpm with a series of applied potentials (30–130 mV) to determine steady‐state current densities. The Tafel slope was plotted against the average current density over the last 30 s. Chronopotentiometry (CP) was used for the electrolysis experiments (unless otherwise stated) and reaction rate studies using deuterated reagents. All electrochemical data in this work are reported without *iR*‐compensation. For the Tafel analysis, the data were post‐corrected for the uncompensated solution resistance (R_u_) to isolate the intrinsic catalytic kinetics. The R_u_ value was determined prior to the measurements via electrochemical impedance spectroscopy (EIS), and potentials were manually adjusted using the relation: *E*_comp_ = *E*_measured_ – *i*R_u_.

### 4.3 Physical Characterizations

Scanning electron microscopy (SEM) was performed using a Tescan Mira 3 LMU FEG microscope operated at 10 kV and equipped with an energy‐dispersive x‐ray spectroscopy (EDX) silicon drift detector (Bruker Quantax 200). X‐ray photoelectron spectroscopy (XPS) measurements were carried out using a Kratos AXIS SUPRA instrument with a monochromatic Al‐Kα x‐ray source (225 W) under a vacuum of 10^−^^8^ Torr. The analysis was conducted with an acceleration voltage of 15 kV, a filament current of 15 mA, and a pass energy of 20 eV to obtain high‐resolution spectra. The C 1s peak (284.8 eV) was used as a calibration reference. X‐ray diffraction (XRD) analysis was performed using a Bruker D8 ADVANCE diffractometer in locked‐coupled mode with an x‐ray source operating at 40 kV and 40 mA. Focused ion beam (FIB) analysis was conducted using a Carl Zeiss Crossbeam 550 system with an SE (In‐Lens) detector at an acceleration voltage of 5 kV. Transmission electron microscopy (TEM) was performed using a Thermo Fisher Scientific Spectra 300 microscope operated at 300 kV for cross‐sectional analysis of the Pd_m_ with secondary metal layer, and STEM and EDX mapping were conducted on the FIB‐prepared samples (Figure 2d). In addition, TEM, STEM, and EDX mapping of the secondary metals physically detached from the Pd_m_ were performed using a Titan Themis (T4) double Cs‐corrected TEM (image/probe Cs).

### 4.4 Pd_m_ and Secondary Metal Layer Preparation

Prior to use, the Pd_m_ was cleaned by immersing in a solution of distilled water, 35% hydrogen peroxide, and 60% nitric acid (2:1:1 ratio) for 15 min on both sides. They were then sequentially rinsed with distilled water, ethanol, and acetone, and dried at room temperature. It was then stored in a sealed container until use.

A stock electrolyte solution was prepared by adjusting the pH of nitric acid (0.1 M) to 3.6 using a 1 N sodium hydroxide solution. The precursor solution was prepared by adding either palladium (II) nitrate hydrate or a rhodium (III) nitrate solution to a portion of the stock solution to achieve a total metal concentration of 5 mM. For the binary metals, the total metal concentration was identical to the equimolar ratio of Pd to Rh. These solutions were used as electrodeposition baths. The cleaned bare Pd_m_ was employed as the working electrode (1.77 cm^2^), with a Ag/AgCl reference electrode and a Pt coil counter electrode.

Electrodeposition was then carried out in a custom‐designed electrochemical cell by applying a constant potential of +0.15 V and −0.15 V until a total charge of 15 C had passed (Figure 2). After the deposition, it was rinsed, dried, and stored in a sealed container.

Based on the measurement of the weight difference by microbalance, the average metal loading was 2 mg cm^−2^ for the Pd electrodeposit and that of bimetallic PdRh, whereas that of the Rh electrodeposit was 0.6 mg cm^−2^. Pd particles from its electrodeposit were prepared by detaching them from the Pd_m_ via sonication in ethanol, followed by centrifugation at 10,000 rpm and drying at 85°C. An equivalent amount of Pd particles was used for Pd loading for onto the e‐Pd/Pd_m_, as shown in Figure 5.

### 4.5 Electrochemical Hydrogenation via ePMR

Electrochemical hydrogenation reactions were performed using an ePMR setup. The aqueous compartment contained 35 mL of 1 M H_2_SO_4_ in an open configuration, whereas the organic compartment contained 35 mL of 100 mM 1‐hexyne in heptane with bibenzyl as the internal standard in a closed configuration. Aliquots of 100 µL were withdrawn periodically during the reaction. Both compartments were stirred at approximately 300 rpm. In general, CP was performed at a current density of −24 mA cm^−2^ for 12 h, corresponding to a total charge of 1,858 C (unless otherwise stated). CA was complementarily conducted at −0.3 V for 12 h for comparison. As a control experiment, Ar gas was purged near the Pd_m_ surface in the organic compartment to remove the hydrogen gas evolved at the Pd surface (Figure 4). To prevent solvent evaporation, Ar gas flow (30 mL min^−1^) was passed through a pre‐bubbler filled with the same organic solution prior to entering the reactor.

### 4.6 Product Analysis

The reaction products in the organic phase were analyzed by GC‐MS (Agilent 8890 GC coupled with a 5977C mass selective detector HP‐5MS column). Bibenzyl (22 mM in the organic reaction mixture) was added as an internal standard for quantitative analysis. Prior to analysis, the organic‐phase samples were diluted 10‐fold with acetone. A 1 µL aliquot of the diluted sample was injected at an inlet temperature of 250°C using a split ratio of 50:1. Helium was used as the carrier gas at a flow rate of 0.8 mL min^−^^1^. The GC oven temperature was initially maintained at 34°C for 4 min, followed by heating to 40°C at a rate of 2°C min^−^^1^ and subsequently to 300°C at a rate of 40°C min^−^^1^, without additional holding periods. The mass spectrometer was operated in scan acquisition mode with a solvent delay of 2.3 min and a mass scan range of m/z 50–500. Calibration curves were constructed by comparing the signal area of the various concentrations of standards vs. a fixed amount of internal standard, slope being the relative response factor for the plot of the ratio of signal area of analyte over internal standard vs. concentration of standards, typically 5‐points in the range of 0.5–20 mM. All the analyte samples (vide supra) were within the calibration curve and further diluted for the measurement if needed. The calibration curves were periodically updated as the column state changes upon use.

The acquired gas chromatogram shows distinct peaks for 1‐hexene, *n*‐hexane, and 1‐hexyne, whereas the *trans*‐2, *cis*‐3, and *trans*‐3‐hexene isomers are indistinguishable. Thus, all hexene isomers, except 1‐hexene, were quantified as a combined group.

In this work, relative concentration refers to the relative molar distribution of all hydrocarbon species of reactant and products analyzed by GC‐MS mentioned above. Relative concentrations of products were calculated using the following equation:

$\mathrm{Relative}\;\mathrm{concentration}\;\left(\%\right)=\frac{C_{i}}{\sum C_{j}}\times 100$ Where ***C***_***i***_ is the concentration of compound *i* and ∑ ***C***_***j***_ is the sum of the concentrations of all quantified products. Selectivity of products could be defined by the ***C***_***i***_ over ∑ ***C***_***j***_ but without the reactant, 1‐hexyne. Quantitative discussion of selectivity was avoided in this work but qualitatively.

Note that relative concentration of the reactant, 1‐hexyne, was omitted throughout this work since its amount does not directly relate to conversion. Rather, it should be derived from each molar composition, which refers to molar distribution of all hydrocarbons over initial concentration of the 1‐hexyne reactant (***C***_1 − ***hexyne***,  0_) quantified by GC‐MS:

$$\mathrm{Composition}\;\left(\%\right)=\frac{C_{i}}{C_{1-\mathrm{hexyne},\;0}}\times 100$$

Note that most of the bar graphs for composition add up to roughly 100% but not exactly due to either measurement error or unidentified side reaction of 1‐hexyne (other than HER). Thus, the conversion of 1‐hexyne (consumed amount) can be defined as follows:

$$\mathrm{Conversion}\;\left(\%\right)=\frac{C_{1-\mathrm{hexyne},\;0}-C_{1-\mathrm{hexyne},\;t}}{C_{1-\mathrm{hexyne},\;0}}\times 100$$

where ***C***_1 − ***hexyne***,  ***t***_ is the concentration of 1‐hexyne after the electrolysis for time period, *t*, quantified by GC‐MS.

Faradaic efficiency (FE) of electrochemical hydrogenation of products was calculated as

$$\begin{array}{cc} & \mathrm{FE}\;\mathrm{of}\;\mathrm{products}\;\left(\%\right)\\ & =\frac{\left\{\left(n_{\mathrm{hexenes}}\times 2H)+(n_{\mathrm{hexane}}\times 4H\right)\right\}\times F}{i\times t}\;\times 100 \end{array}$$

where ***n***_***hexenes***_ and ***n***_***hexane***_ are the number of moles of hexenes and *n*‐hexane produced, respectively, ***i*** is the applied current (A), ***t*** is the electrolysis duration (s), and ***F*** is the Faraday constant (96,485 C mol^−1^).

H_2_ gas evolution quantification in the organic compartment was performed by GC with a thermal conductivity detector (GC‐TCD; SHIMADZU GC‐2030, Rtx‐Wax column). The column was operated with an initial temperature of 60°C and a 7 min hold. The setup was identical to that of the ePMR system, except that an Ar flow (20 sccm) was introduced at a position distant from the Pd_m_. The outlet gas was sampled from the reactor headspace, followed by direct injection into the GC (inline mode); measurements were conducted every hour. Organic product analysis (GC‐MS) and H_2_ gas quantification (GC‐TCD) were performed independently in separate experiments. The FE of H_2_ generated in the organic phase was calculated according to the

$$\mathrm{FE}\;\mathrm{of}\;H_{2}\;\mathrm{gas}\;\;\left(\%\right)=\frac{n\times 2\times F}{i\times t}\;\;\times 100\;$$

Here,

$$n=\frac{P\times \left(H_{2}\;\mathrm{vol}.\%/100\right)\times \dot{V}\times t}{R\times T}$$

where ***n*** is the number of moles of H_2_ detected (mol), ***P*** is the total pressure (1 atm), ***H***_2_ ***vol***.% is the volume percent of H_2_ detected by GC‐TCD, $\dot{V}$ is the total volumetric flow rate (L s^−1^), ***R*** is the gas constant (0.0821 atm L mol^−1^ K^−1^) and ***T*** is the reaction temperature (298.5 K).

### 4.7 Chemical Hydrogenation With H_2_ Gas

The electrochemical compartment was kept empty and sealed with a lid, whereas the organic compartment was filled with the same solution used in the ePMR setup. High‐purity H_2_ was introduced at a flow rate of 30 sccm. To minimize the solution evaporation, the gas flow was passed through a pre‐bubbler containing the reaction solution before entering the reactor. The H_2_ bubbler was introduced near the Pd_m_ and then directed through a –10°C condenser before venting. The organic compartment was stirred at 300 rpm and the reaction was conducted for 6 h without intermediate product sampling. The efficiency of H_2_ gas utilization was calculated as:

$$\begin{array}{cc} & \mathrm{Efficiency}\;\mathrm{of}\;H_{2}\;\mathrm{gas}\;\mathrm{utilization}\;\;\left(\%\right)\\ & =\frac{n_{H_{2},\;\mathrm{consumed}\;\mathrm{for}\;\mathrm{products}}}{n_{H_{2},\;\mathrm{total}\;\mathrm{feed}}}\times 100,\\ & =\frac{\left(n_{\mathrm{hexenes}}\times 1H_{2})+(n_{\mathrm{hexane}}\times 2H_{2}\right)}{P\times V_{H_{2}}/\left(R\times T\right)}\;\times 100 \end{array}$$

with

$$\;V_{H_{2}}={\dot{V}}_{H_{2}}\times t$$

where ***n***_***hexenes***_ and ***n***_***hexane***_ are the number of moles of hexenes and *n*‐hexane produced (mol), ${\dot{V}}_{H_{2}}$ is the volumetric flow rate of H_2_ fed (L s^−1^), ***t*** is the hydrogenation time (s), $V_{H_{2}}$ is the total volume of H_2_ fed (L).

## Author Contributions

**Samuel J. Shin**: conceptualization, investigation, Writing – original draft, Writing – review and editing, methodology, validation. **Su Bin Roh**: data curation, methodology, writing – review and editing, investigation, formal analysis. **Hyun‐Tak Kim**: investigation. **Kwan‐Young Lee**: validation. **Soo Min Kim**: investigation. **Ji Hoon Park**: project administration, conceptualization, investigation, supervision. **Minju Park**: conceptualization, writing – original draft, writing – review and editing, investigation, visualization, supervision.

## Conflicts of Interest

The authors declare no conflicts of interest.

## Supporting information




**Supporting File**: advs78365‐sup‐0001‐SuppMat.docx.

## Data Availability

The data that support the findings of this study are available from the corresponding author upon reasonable request.
