# Supplementary material for: Deciphering Electrochemical Generation, Transport, and Reactivity of Hydrogen Species in the Electrochemical Palladium Membrane Reactor
Source: Adv Sci (Weinh). 2026 Oct 18:e78365. Online ahead of print. doi: 10.1002/advs.78365 (PMC13677042; doi:10.1002/advs.78365)
Supplement: Supplementary file 1 [file ADVS-9999-e78365-s001.docx]

Supplementary Information

**Deciphering Electrochemical Generation, Transport, and Reactivity of Hydrogen Species in Electrochemical Palladium Membrane Reactor**

Samuel J. Shin^a,†^, Su Bin Roh^a,b,†^, Hyun-Tak Kim^a,c^, Kwan-Young Lee^b^, Soo Min Kim^a^, Ji Hoon Park^a,c,*^, and Minju Park^a,*^

^a^ CO_2_ & Energy Research Center, Korea Research Institute of Chemical Technology (KRICT), Daejeon 34114, Republic of Korea

^b^ Department of Chemical and Biological Engineering, Korea University, Seoul 02841, Republic of Korea

^c^ Department of Advanced Materials and Chemical Engineering, University of Science and Technology (UST), Daejeon 34113, South Korea

^†^ These authors contributed equally

E-mail: [minju@krict.re.kr](mailto:minju@krict.re.kr), [jihpark@krict.re.kr](mailto:jihpark@krict.re.kr)

**Figure S1.** Hydrogenation performance evaluation during a 12 h reaction period for bare Pd_m_, plotted against composition instead of relative concentration **(Figure 1c)**.

**Figure S2.** Electrochemical analysis and hydrogenation performance of 1-hexyne using a bare Pd membrane.(a) CV was performed with a scan rate of 100 mV s^-1^ and the potential range from +1.0 V to –0.6 V. The electrochemical compartment contained 1 M H_2_SO_4_ 35 mL, and the chemical compartment was maintained as empty. Hydrogenation performance and faradaic efficiency on a constant potential electrolysis in a three-electrode configuration of 100 mM 1-hexyne for 3 h, varying (b) organic solvents and (c) applied potentials.

**Figure S3.** Hydrogenation performance and faradaic efficiency of 1-hexyne under varying current densities with identical total charge 1,858 C passed. (a) –73 mA cm^-2^ with sampling every 1 h, (b) –146 mA cm^-2^ with sampling every 30 min.

**Figure S4.** Apparent isotopic effect analysis for hydrogenation of 1-hexyne to 1-hexene under H and D sources with the bare Pd membranes. (a) CA profiles recorded during electrolysis at –0.3 V for 2 h and (b) mass spectra of the resulting 1-hexene and 1-hexene-*d*_2_ obtained from (a). (c) Mass spectra of the resulting 1-hexene and 1-hexene-*d*_2_ obtained from CP profiles recorded during electrolysis at –24 mA cm^-2^ for 2 h.

**Figure S5.** SEM images and corresponding EDX elemental maps of electrodeposited Pd catalysts (e-Pd/Pd_m_) prepared at different potentials, before and after hydrogenation coupled with electrolysis at –0.3 V for 12 h.

e-Pd/Pd_m_ before reaction: prepared at (a) +0.15 V and (b) –0.15 V. e-Pd/Pd_m_ after reaction: prepared at (c) +0.15 V and (d) –0.15 V.

**Figure S6.** SEM images and corresponding EDX elemental maps of electrodeposited Rh catalysts (e-Rh/Pd_m_) prepared at different potentials, before and after hydrogenation coupled with electrolysis at –0.3 V for 12 h.

e-Rh/Pd_m_ before reaction: prepared at (a) +0.15 V and (b) –0.15 V. e-Rh/Pd_m_ after reaction: prepared at (c) +0.15 V and (d) –0.15 V.

**Figure S7.** XPS spectra of the Pd 3d region for electrodeposited Pd catalysts (e-Pd/Pd_m_) prepared at different potentials, before and after hydrogenation coupled with electrolysis at –0.3 V for 12 h.e-Pd/Pd_m_ prepared at (a) +0.15 V and (b) –0.15 V.

**Figure S8.** XPS spectra of the Rh 3d region for electrodeposited Rh catalysts (e-Rh/Pd_m_) prepared at different potentials, before and after hydrogenation coupled with electrolysis at –0.3 V for 12 h. e-Rh/Pd_m_ prepared at (a) +0.15 V and (b) –0.15 V.

**Figure S9.** SEM images and corresponding EDX elemental maps of electrodeposited PdRh alloy catalysts (e-PdRh/Pd_m_) prepared at different potentials, before and after hydrogenation coupled with electrolysis at –0.3 V for 12 h.

e-PdRh/Pd_m_ before reaction: prepared at (a) +0.15 V and (b) –0.15 V. e-PdRh/Pd_m_ after reaction: prepared at (c) +0.15 V and (d) –0.15 V.

**Figure S10.** XPS spectra of Pd 3d and Rh 3d regions for electrodeposited PdRh alloy catalysts (e-PdRh/Pd_m_) prepared at different potentials, before and after hydrogenation coupled with electrolysis at –0.3 V for 12 h. Pd 3d spectra of e-PdRh/Pd_m_ prepared at (a) +0.15 V and (b) –0.15 V. Rh 3d spectra of e-PdRh/Pd_m_ prepared at (c) +0.15 V and (d) –0.15 V.

**Figure S11.** (a) STEM images and corresponding EDX elemental maps of e-PdRh aprticles deteched from e-PdRh/Pd_m_ prepared at +0.15 V, and (b) EDX line profile along the indicated arrow.

**Figure S12.** XRD patterns of e-Pd particles deteched from e-Pd/Pd_m_ prepared at +0.15 V.

**Figure S13.** CP profiles and GC chromatograms during hydrogenation coupled with electrolysis at –24 mA cm⁻^2^ for 12 h using bare Pd and electrodeposited Pd, Rh, and PdRh membranes (prepared at +0.15 V). (a) CP profiles of the Pd membranes during electrolysis. (b–e) GC chromatograms collected every 3 hours during hydrogenation with (b) bare Pd_m_, (c) e-Pd/Pd_m_, (d) e-Rh/Pd_m_, and (e) e-PdRh/Pd_m_, respectively.

**Figure S14.** Hydrogenation performance evaluation during a 12 h reaction period for (a) e-Pd/Pd_m_, (b) e-Rh/Pd_m_, and (c) e-PdRh/Pd_m_, plotted against composition instead of relative concentration **(Figure 3a-c)**.

**Figure S15.** Hydrogenation performance evaluation when (a) H_2_ gas feeding without external bias and (b) Ar gas purging with external bias, plotted against composition instead of relative concentration **(Figure 4b and 4d-f)**.

**Figure S16.** Setup illustrations of the ePMR configurations and their corresponding hydrogenation performance and faradaic efficiency for the investigation of distance of e-Pd to Pd_m_. (a) Setup with dispersed e-Pd particles detached from e-Pd/Pd_m_ prepared at +0.15 V, and (b) e-Pd/Pd_m_ placed far from the hydrogen formation site using a three-compartment cell. Hydrogenation performance and faradaic efficiency of 1-hexyne (c) and (d) correspond to setups (a) and (b), respectively. Aq., aqueous phase; Org., organic phase.
